# Malignant Hyperthermia: Report of Two Cases with a Neglected Complication in Cardiac Surgery

**Published:** 2017-10

**Authors:** Mahdi Neshati, Manizheh Azadeh, Parinaz Neshati, Tyrone Burnett, Ryan Saenz, Bahman Karbasi, Ghader Shahmohammadi, Eskandar Nourizadeh, Mohsen Rostamzadeh

**Affiliations:** 1 Department of Cardiothoracic Surgery, Ostad Aalinasab Hospital, Tabriz, Iran.; 2 Institute of Clinical Evaluative Sciences (ICES), Sunnybrook Hospital, University of Toronto, Toronto, Canada.; 3 Greater Houston Interventional Pain Associates, Houston, USA.; 4 Department of Anesthesiology, University of Texas Health Science Center, Houston, USA.

**Keywords:** *Cardiac surgical procedures*, *Malignant hyperthermia*, *Cardiopulmonary bypass*, *Postoperative care*

## Abstract

Malignant hyperthermia (MH) can develop after contact with volatile anesthetics (halothane, enflurane, isoflurane, sevoflurane, and desflurane) as well as succinylcholine and cause hypermetabolism during anesthesia, which is associated with high mortality when untreated. Early diagnosis and treatment could be life-saving. During cardiac surgery, hypothermia and cardiopulmonary bypass make the diagnosis of MH extremely challenging compared with other settings such as general surgery.

We herein report 2 cases of MH, graded as “very likely” or “almost certain” based on the MH clinical grading scale. A 14-month-old infant and a 53-year-old male underwent surgery for severe pulmonary valve stenosis and mitral valve replacement, respectively. Both of them were extubated on the operation day, but they deteriorated with the development of high-grade fever, hypotension, renal failure, and acidosis. The first case had muscle spasms. Unfortunately, the delayed symptoms of MH in the early postoperative course were not diagnosed in these 2 cases, which caused permanent neurologic damage in the first case and death in the second one. However, the infant was discharged from the hospital after 2 months.

## Introduction

Malignant hyperthermia (MH) is a potentially lethal hypermetabolic syndrome which can develop after using volatile anesthetics (halothane, enflurane, isoflurane, sevoflurane, and desflurane) and succinylcholine. Susceptibility to MH is an autosomal dominant inherited disorder. The overall incidence of MH is estimated to be between 1 in 5000 and 1 in 200000 anesthesia procedures. The prevalence of MH susceptibility could be as high as 1 in 3000 in the general population. Because of the widespread use of volatile anesthetics in cardiac surgery, MH remains a relevant concern.^1^^-^^5^

MH primarily affects the calcium hemostasis of skeletal muscles. Contact with trigger substances leads to a massive calcium release from the sarcoplasmic reticulum in response to a defective ryanodine receptor. Increased intracellular calcium levels activate contractile filaments and stimulate the cellular energy turnover. Dramatic increases in oxygen consumption and CO_2_ production are followed by a hugely increased production of lactic acid. The ongoing stimulation finally leads to a breakdown of the cellular metabolism and integrity, and a massive release of muscular enzymes.^1^^, ^^3^^, ^^5^

The syndrome includes hemodynamic changes to meet the increased oxygen demand. Expiratory and venous PCO_2_ values increase and lactic acidosis develops with mixed metabolic and respiratory acidosis. The most frequent and earliest sign of MH crisis is an unexpected tachycardia associated with an unexplained rise (over minutes to hours) in end-tidal CO_2_, the most sensitive indicator of potential MH.^3^^, ^^4^ The early manifestations include sinus tachycardia, unstable blood pressure, and tachypnea. The skin becomes mottled with cyanotic areas and patches of bright red flushing. Most patients develop a rigor mortis-like stiffness of all skeletal muscles. Temperature increases as a result of the muscle contracture, which is a relatively late finding. Other late clinical manifestations include hyperkalemia, grossly elevated blood creatine phosphokinase levels, grossly elevated blood myoglobin levels, dark-colored urine due to myoglobinuria (which can lead to renal failure), severe cardiac arrhythmias, cardiac arrest, and disseminated intravascular coagulation.^1^^, ^^2^^, ^^5^^-^^8^ The definite diagnosis of MH susceptibility can be made by the caffeine halothane contracture test with muscle biopsy.^9^^, ^^10^

During cardiopulmonary bypass (CPB), these symptoms are often obscured, in part because the MH-induced increase in cellular metabolism may be mitigated by the imposed hypothermia. Patients undergoing cardiac surgery often have more significant underlying medical conditions than other surgical patients do. Perioperative complications are, therefore, attributed to these underlying problems, and an MH diagnosis is, thus, often delayed or neglected. Timely treatment is essential, so early symptoms must be identified and interpreted appropriately.^5^^, ^^11^^-^^21^

When MH is untreated, mortality occurs in 80% of cases due to multi-organ failure while treatment with dantrolene reduces mortality to 5%.^1^^, ^^3^^-^^5^^, ^^22^ With a 50% probability of MH recrudescence, treatment with dantrolene is recommended to continue for 36 to 48 hours.^1^^, ^^3^^, ^^4^^, ^^23^ Hypothermia was the main treatment before dantrolene was used and currently it is considered an additional therapy to datrolene.^1^^-^^5^

## Report of the Cases

We reviewed the first 48-hour postoperative period in 959 patients with cardiac surgery performed by a cardiovascular surgeon, between October first 2007 and October first 2014 in Ostad Aalinasab Hospital, Tabriz, Iran. Seventy-two critically ill patients who were hemodynamically unstable in the first 48 hours postoperatively were selected. Patients with known causes of unstable hemodynamics were excluded from the study. The MH clinical grading scale (Table 1) was calculated for the patients. Our inclusion criteria were based on the MH clinical grading scale, and we selected patients with “very likely” or “almost certain” MH. 

The MH clinical grading scale estimates the qualitative likelihood of MH in a given patient without using a specific diagnostic test and is based on points assigned to specific abnormal signs and laboratory findings (rigidity, muscle breakdown, respiratory and metabolic acidosis, increased temperature, and cardiac manifestations) during an acute anesthetic reaction. Points were assigned when they were judged by the anesthesiologist to be inappropriate for the patient’s underlying medical condition, anesthetic technique, and surgical procedure.^7^^, ^^8^^, ^^22^

Three patients had critically ill condition after surgery which remained undetermined after workup. Two cases from these 3 cases could be graded as “very likely” or “almost certain” MH. The clinical course and laboratory findings during anesthesia and the early postoperative period of these 2 cases are presented. 

***Case # 1***

A 14-month-old infant was candidated for surgery with the diagnosis of severe pulmonary valve stenosis due to annulus hypoplasia. Past medical history was negative for other diseases. The patient was sedated with ketamine and anesthetized with fentanyl, midazolam, and pancuronium. Anesthesia was maintained with atracurium, midazolam, remifentanil infusion, and isoflurane inhalation. Isoflurane was not administered during CPB. CPB was established, and the patient was cooled down to 24 ˚C. Pulmonary valve stenosis was successfully corrected with a transannular patch. At the end of CPB, his blood pressure was 90/45 mm Hg with a heart rate of 130 bpm without receiving inotrope. A low-dose inotrope infusion and furosemide were started in the intensive care unit (ICU) because of low urine output, and he was extubated 5 hours after being transferred to the ICU. After extubation, he showed low PO_2_ and tachypnea. His body temperature was 38.2 ˚C (axillary) at the operative night, and compensated metabolic acidosis appeared gradually with dark urine. Because of the mottling during blood transfusion, the probability of blood incompatibility was considered, which was ruled out with repeat cross-mach. The patient was lethargic on the first postoperative morning. One day after surgery, the patient had tachypnea with low PO_2_ and exacerbated metabolic acidosis. Due to hypotension, the inotrope dose was increased and saline and furosemide infusion were initiated to maintain urine output. Fever increased to 39 ˚C. The patient had suspicious convulsion movements similar to extremity spasm without clonic phase, 40 hours after admission to the ICU. Treatment began with midazolam, and low calcium levels were corrected. Fever increased to 40.3 ˚C (axillary). Intubation was done 48 hours after surgery due to loss of consciousness, metabolic acidosis, and respiratory distress. During intubation, atracurium was infused for to the jaw spasm. Antibiotic therapy with meropenem and metronidazole was started to cover the suspected diagnosis of meningitis or aspiration pneumonia, and acetaminophen was infused at the same time. The patient was cooled with a cooler blanket for 2 days. Gradually, the patient’s metabolic acidosis improved, and his blood pressure and urine output were increased by cooling. He was suffering from jaw spasm. The frequency of convulsions increased when the cooling process was stopped on days 5, 6, and 7 while full-dose phenytoin was unsuccessful in convulsion control. Midazolam was used frequently to control muscular spasm, and the blood calcium level was corrected with a high-dose calcium infusion. Neurological examination and brain computed tomography (CT) scan were negative for any focal finding. Echocardiography revealed normal ventricular function without residual pulmonary valve stenosis or pulmonary regurgitation. Low PO_2_ was continuing in spite of mechanical ventilation. Chest X-ray was negative for pneumonia. There was bilateral pleural effusion, which was exacerbated 1 week later. Blood, tracheal tube, and urine culture were negative. The patient had low-grade fever after 1 week. The last muscular spasm was reported during postoperative day 8.

The patient was transferred to the pediatric ICU. Infection workup was negative. He was weaned off the ventilator and discharged after 2 months while suffering from central nervous system side effects (Table 2 and Table 3).

**Table 1:** Malignant hyperthermia clinical grading score

*Scoring Rule*			
Malignant hyperthermia indicators			
• Review the list of clinical indicators. If any indicator is present, add the points applicable for each indicator while observing the double-counting rule below, which applies to multiple indicators representing a single process.			
• If no indicator is present, the patient’s malignant hyperthermia score is 0.			
Double counting			
• If more than 1 indicator represents a single process, count only the indicator with the highest score*.* Application of this rule prevents double counting when 1 clinical process has more than 1 clinical manifestation.			
• Exception: The score for any relevant indicators in the final category “other indicators” should be added to the total score without regard to double- counting.			
Interpreting the raw score: Malignant hyperthermia rank and qualitative likelihood			
Raw Score Range	Malignant Hyperthermia Rank	Description of Likelihood	
0	1	Almost never	
3–9	2	Unlikely	
10–19	3	Somewhat less than likely	
20–34	4	Somewhat greater than likely	
35–49	5	Very likely	
50+	6	Almost certain	

*Clinical Indicators for Use in Determining the * *Malignant Hyperthermia* * Raw Score*			
**Process I: Rigidity**			
Indicator			Points
Generalized muscular rigidity (in the absence of shivering due to hypothermia, or during or immediately after emer- gence from inhalational general anesthesia)			15
Masseter spasm shortly after succinylcholine administration			15
**Process II: Muscle Breakdown**			
Indicator			Points
Elevated creatine kinase > 20000 U after anesthetics that include succinylcholine			15
Elevated creatine kinase > 10000 U after anesthetics without succinylcholine			15
Cola-colored urine in the perioperative period			10
Myoglobin in urine > 60 µg/L			5
Myoglobin in serum > 170 µg/L			5
Blood/plasma/serum K+> 6 mEq/l (in the absence of renal failure)			3
**Process III: Respiratory Acidosis**			
Indicator			Points
End-tidal pressure of carbon dioxide (PETCO_2_) > 55 mm Hg with appropriately controlled ventilation			15
Arterial carbon dioxide tension (PaCO_2_) > 60 mm Hg with appropriately controlled ventilation			15
PETCO_2_ > 60 mm Hg with spontaneous ventilation			15
Arterial PaCO_2_ > 65 mm Hg with spontaneous ventilation			15
Inappropriate hypercarbia (in anesthesiologist’s judgment)			15
Inappropriate tachypnea			10
**Process IV: Temperature Increase**			
Indicator			Points
Inappropriately rapid increase in temperature (in the anesthesiologist’s judgment)			15
Inappropriately increased temperature > 38.8 °C (101.8 °F) in the perioperative period (in the anesthesiologist’s judgment)			10
**Process V: Cardiac Involvement**			
Indicator			Points
Inappropriate sinus tachycardia			3
Ventricular tachycardia or ventricular fibrillation			3
**Other Indicators That Are Not Part of a Single Process** *			
Indicator			Points
Arterial base excess more negative than -8 mEq/l			10
Arterial pH < 7.25			10
Rapid reversal of malignant hyperthermia signs of metabolic and/or respiratory acidosis with intravenous dantrolene			5

Adapted from [Larach MG, Localio AR, Allen GC, Denborough MA, Ellis FR, Gronert GA, Kaplan RF, Muldoon SM, Nelson TE, Ording H, Rosenberg H, Waud BE, Wedel DJ. A clinical grading scale to predict malignant hyperthermia susceptibility. Anesthesiology 1994;80:771-779.], and [Larach MG, Brandom BW, Allen GC, Gronert GA, Lehman EB. Cardiac Arrests and Deaths Associated with Malignant Hyperthermia in North America from 1987 to 2006. A Report from the North American Malignant Hyperthermia Registry of the Malignant Hyperthermia Association of the United States. Anesthesiology 2008;108:603-611.]. Published with permission from Lippincott Williams& Wilkins.

* These should be added without regard to double counting.

**Table 2:** Blood gas analysis and clinical course of Case 1

Post op. day	Time	BP (mm Hg)	HR (/min)	BT(˚C)	RR vent/ RR spont (/min)	PH	HCO_3 _(mmol/lit)	BE (mmol/lit)	PCO_2 _(mm Hg)	PO_2 _(mm Hg)	O_2_Sat (%)	FiO_2 _(%)	K+ (mmol/lit)		
The day of operation	09:12	90/45	120	37.0	-	7.51	19.2	-2.7	24	426	100	100	3.6	Anesthesia	
	10:50	41	-	32.0	-	7.49	19.5	-4.5	24	375	100	70	2.3		
	11:30	42	-	30.0	-	7.51	20.3	-4.3	24	259	100	55	4.3		
	12:10	42	-	35.0	-	7.40	16.8	-7.7	27	243	100	75	4.0		CPB+filter
	12:31	72/35	120	-	-	7.46	21.3	-1.9	30	366	100	100	3.6		
	12:53	90/45	130	-	-	7.45	25.0	1.2	36	83	97	-	2.9		
	13:14	80/42	125	-	-	7.43	25.9	1.5	39	105	98	-	3.3		
	13:43	90/60	150	-	25/0	7.43	25.2	0.9	38	180	100	100	3.2		Extubation
	18:21	95/50	150	36.5	25/14	7.43	21.9	-1.8	33	61	92	50	4.1		
1	00:31	100/60	130	36.5	-/42	7.45	19.5	-3.4	28	55	90	-	5.0		
	06:53	115/65	180	38.0	-/58	7.47	20.4	-2.2	28	71	95	30	4.8		
	12:13	115/70	110	-	-/55	7.40	13.6	-9.3	22	59	90	35	5.6		
	19:33	90/55	140	39.0	-/40	7.40	19.8	-4.2	32	91	97	35	5.0		First spasm
2	01:30	80/45	150	38.5	-/45	7.36	17.5	-6.7	31	58	88	35	4.2		
	06:14	70/35	150	38.5	-/45	7.28	19.7	-6.8	42	55	84	-	3.8		
	08:43	100/55	185	40.3	-/68	7.42	15.6	-	24	63	85	-	3.1		Reintubation
	09:56	75/35	180	39.5	25/10	7.41	21.6	-2.4	34	67	93	100	3.8		
	12:25	75/35	180	40.2	22/8	7.47	18.9	-3.2	26	74	96	80	4.0		
	17:33	60/30	140	36.2	20/2	7.34	17.3	-7.4	32	61	89	50	3.5	Surface cooling	
	23:11	60/40	145	37.0	20/1	7.34	17.3	-7.3	32	73	93	50	3.6		
3	06:32	70/40	165	37.3	15/9	7.41	18.4	-4.9	29	83	96	50	4.0		
	11:37	70/30	165	38.5R	15/25	7.44	19.0	-3.8	28	66	93	40	4.3		

op, Operation; BP, Blood pressure (the mean arterial pressure during cardiopulmonary bypass); HR, Heart rate; BT, Body temperature (the nasopharyngeal temperature during anesthesia or axillary temperature during intensive care unit stay); RRvent/RRspont, Ventilation rate/spontaneous respiratory rate; BE, Base excess; PCO_2_, Partial pressure of carbon dioxide; PO_2_, Partial pressure of oxygen; O_2_ Sat, Oxygen saturation; Fio_2_, Fraction of inspired oxygen; CPB

+ filter, Cardiopulmonary bypass with hemofiltration; R, Rectal temperature

***Case # 2***

A 53-year-old male was candidated for mitral valve replacement due to severe calcified mitral stenosis with a left ventricular ejection fraction of about 55%. History was negative for other chronic diseases. In coronary angiography and right-heart catheterization, moderate stenosis was reported in the mid-portion of the left anterior descending artery, with no need for intervention. The pulmonary artery pressure was 60/35 mm Hg. During the operation, anesthesia was maintained with an infusion of atracurium, midazolam, and remifentanil as well as with an inhalation of isoflurane. Isoflurane was not used during CPB. After establishing CPB, the patient was cooled down to 30 ˚C. The mitral valve was replaced with a CarboMedics valve (No. 29). The patient had a systolic pressure > 100 mm Hg at the end of CPB; however, due to subsequent hypotension, an epinephrine infusion was started. He was transferred to the ICU in hypotensive status with transient premature ventricular contractions while receiving epinephrine. The patient was extubated after 7 hours while he had compensated metabolic acidosis. He was conscious on the first morning after surgery and was out of bed without abnormal physical finding. He gradually developed tachypnea and suffered severe pulmonary edema in the afternoon. His heart rate increased to 150 bpm with atrial fibrillation rhythm and hypotension. He developed metabolic acidosis with a decreased urine output. Transthoracic echocardiography revealed preserved left and right ventricular functions. The peak and mean transprosthetic mitral valve gradients were 38.4 mm Hg and 15.8 mm Hg, respectively, without paravalvular leakage. Fluoroscopy demonstrated normal mechanical valve function, and the measured gradient was thought to be related to tachycardia. In postoperative electrocardiogram (ECG), T-wave inversion was detected in leads II and III and AVF without ST-segment elevation. Due to severe respiratory distress and impaired consciousness, the patient was intubated on the first postoperative night. Atracurium was administered during intubation. In spite of receiving high-dose inotrope and fluids, the patient was hypotensive and anuric with severe metabolic acidosis and low PO_2_. The patient’s chest was opened in the ICU to rule out cardiac tamponade, which was negative with good ventricular contraction in a hyperkinetic status. On the second postoperative morning, the patient showed severe metabolic acidosis with hypotension, anuria, and 40.5 ˚C fever (axillary). The patient was transferred to the operating room in pre-arrest state. Anesthesia was maintained by the infusion of atracurium, midazolam, and remifentanil and CBP was established immediately. After the beginning of CPB, transesophageal echocardiography was done and revealed a suspected mass at the ventricular side of the mechanical mitral valve. The contraction of the left and right ventricles was reduced significantly. With suspected mechanical mitral valve malfunction, aortic cross-clamp and cardiac arrest were performed, body temperature was reduced to 28 ˚C, and the mechanical mitral valve was exposed. The function of the mechanical valve was normal, and there was about 10% to 15% paravalvular leakage. The exploration of the mentioned suspected mass became possible by removing the prosthetic valve: There were no abnormal findings inside the left ventricle. The vein graft was anastomosed to the left anterior descending artery due to moderate stenosis, and the mechanical valve was replaced for a second time. During CPB, the patient showed severe metabolic acidosis with lactate > 15 mmol/lit not eliminated by CPB. An intra-aortic balloon pump was inserted, and CPB was ended with high-dose epinephrine. The patient was transferred to the ICU with an open sternum and severe hypotension. In spite of ventilation with high FiO_2_, PO_2_ was low. Due to anuria and severe acidosis at the end of the reoperation day, continuous venovenous dialysis was initiated. Hypocalcemia developed on the second postoperative day continued during the following days. Peritoneal dialysis was performed at the end of the third postoperative day without any improvement in the patient’s condition. Sedation was discontinued on the third postoperative day owing to deep coma status. Because of jaw spasm, the mouth suction was difficult. Creatine phosphokinase (CPK) enzyme and creatine phosphokinase-MB (CPK-MB) enzyme were measured 43500 U and 12100 U, respectively, with hypoglycemic crisis on days 3, 4, and 5 after the operation. Despite severe hypotension, the extremities were warm with palpable pedal pulses. The patient died after 5 days due to multi-organ failure (Table 3 and Table 4).

**Table 3:** Malignant hyperthermia clinical grading score of the 2 cases

Clinical Indicator	Case 1	Case 2
Rigidity	15	
Elevated CPK		15
Cola-color urine	10	
Tachypnea	10	10
Temperature increase	10	10
Tachycardia	3	3
Arterial base excess < -8 mEq/l	10	10
PH < 7.25		10
Score	58	58
Malignant hyperthermia likelihood	Almost certain	Almost certain

CPK, Creatine phosphokinase enzyme

**Table 4:** Blood gas analysis and clinical course of Case 2

Post op. day	Time	BP (mm Hg)	HR (/min)	BT (˚C)	RR vent/ RR spont (/min)	PH	HCO_3_ (mmol/lit)	BE (mmol/lit)	PCO_2_ (mm Hg)	PO_2_ (mm Hg)	O_2_Sat (%)	FiO_2_ (%)	K^+^ (m mol/ lit)		
The day of operation	08:35	80/50	75	-	-	7.42	22.1	-1.7	34	383	100	100	3.2	Anesthesia	CPB+ filter
	09:35	53	-	30.0	-	7.49	23.0	-1.8	28	278	100	60	4.0		
	10:25	75	-	30.0	-	7.48	20.7	-4.0	26	272	100	60	6.3		
	11:01	52	-	37.0	-	7.39	21.8	-2.8	36	326	100	90	5.0		
	11:51	90/55	80	-	-	7.36	20.3	-4.7	36	260	100	100	3.9		Extubate
	12:32	70/45	70	-	11/0	7.29	19.7	-6.5	41	147	99	100	3.1		
	18:57	100/65	70	36.0	0/13	7.30	18.2	-7.6	37	105	97	40	4.3		
1	08:00	125/70	95	37.0	-/26	-	-	-	-	-	93	-	-		Reintubate
	12:00	100/55	95	36.5	-/20	-	-	-	-	-	95	-	-		
	17:08	105/60	150	-	-/20	7.26	16.2	-10.0	36	52	80	40	4.3		
	21:32	85/40	80	-	0/30	7.25	18.0	-8.6	41	62	87	-	4.5		Chest was opened
	23:12	50/20	140	-	12/8	7.33	18.5	-6.8	35	90	96	90	4.6		
2	00:25	30/10	140	-	12/8	7.11	15.9	-13.0	50	50	69	70	3.6		
	06:58	20/10	80	40.5	-	7.26	13.3	-12.9	31	80	93	100	3.9		
	08:18	40/20	120	38.5	-	7.15	17.1	-11.0	49	51	73	100	3.8	Anesthesia	
	08:48	30	-	36.0	-	7.28	22.6	-3.8	48	416	100	90	3.2		
	09:41	38	-	28.0	-	7.29	16.8	-10.8	31	301	100	60	3.0		
	10:10	38	-	28.0	-	7.44	18.1	-7.5	24	252	100	55	3.0		
	11:05	40	-	31.0	-	7.45	14.7	-9.9	20	316	100	70	3.4		
	11:57	40	-	31.5	-	7.41	14.8	-11.1	22	342	100	75	3.9		
	12:29	44	-	36.0	-	7.35	14.9	-9.8	27	341	100	80	4.0		
	13:12	42	-	35.0	-	7.34	14.0	-10.7	26	366	100	80	3.4		
	14:10	40/20	105	-	-	7.25	14.5	-11.7	33	66	89	100	2.9		IABP
	15:16	20/10	130	-	12/0	7.18	16.0	-11.6	43	50	74	100	3.4		
	18:24	45/20	125	-	16/0	7.31	16.6	-8.8	33	50	81	90	3.8		
	21:41	55/25	135	-	12/2	7.29	14.4	-11.0	30	47	77	90	4.0	Dialysis	
	23:01	65/30	140	36.5	13/0	7.28	12.7	-12.6	27	55	84	100	4.2		
3	00:49	75/40	140	-	13/12	7.27	12.4	-13.1	27	58	85	100	3.9		
	03:30	65/30	140	-	-	7.22	11.5	-14.8	28	57	82	-	4.2		
	07:02	55/25	130	36.5	13/9	7.29	13.5	-11.9	28	62	88	90	4.6		
	12:59	50/35	140	37.0	12/8	7.41	19.0	-4.9	30	60	91	80	5.5		
	18:00	55/20	140	37.3	10/18	7.32	18.0	-7.4	35	56	86	80	5.9		
	22:17	75/40	90	-	10/11	7.41	17.1	-6.6	27	65	93	80	6.8		

op, Operation; BP, Blood pressure (the mean arterial pressure during cardiopulmonary bypass); HR, Heart rate; BT, Body temperature (the nasopharyngeal temperature during anesthesia or axillary temperature during intensive care unit stay); RRvent/RRspont, Ventilation rate/spontaneous respiratory rate; BE, Base excess; PCO_2_, Partial pressure of carbon dioxide; PO_2_, Partial pressure of oxygen; O_2_ Sat, Oxygen saturation; Fio_2_, Fraction of inspired oxygen; CPB

+ filter, Cardiopulmonary bypass with hemofiltration; IABP, Intra-aortic balloon pump

## Discussion

Regarding the extensive use of anesthetic medications that trigger MH in cardiac surgery, it seems that MH has a higher incidence in patients undergoing cardiac surgery than with anesthesia in other types of surgeries. Despite this issue and the high number of cardiac surgeries, the reported numbers of MH after cardiac surgery in the literature are disproportionately low. In a systematic review performed by Meterlein et al.,^5^ only 14 cases of MH were detected after cardiac surgery in electronic search of the literature until 2010. It seems that many cases of MH are undiagnosed and not reported after cardiac surgery. To prevent the underdiagnosis of MH, there should be a special effort to discover and find possible cases of MH after cardiac surgery.

In our study, we tried to detect cases with high suspicion of MH. We used the MH clinical grading scale to determine the clinical MH likelihood and selected patients with the highest likelihood of MH. The clinical grading scale has served as a clinical case definition for the MH syndrome in multiple research studies, including those validating the North American caffeine halothane contracture test and the in vitro contracture test (European Malignant Hyperthermia Group).^8^^-^^10^^, ^^21^^-^^24^

Among 959 cardiac surgeries, we detected 2 cases with the highest MH probability according to the MH clinical grading scale. The real incidence of MH may be obscured in cardiac surgery. While a heterogeneous picture of symptoms in the CPB setting makes the early diagnosis of MH difficult, some cases with a negative outcome may not have been published, thus creating a significant reporting bias.^5^

A considerable finding in our cases is that both of them were transferred to the ICU with acceptable hemodynamic status and high-grade fever and other symptoms of MH developed later in the ICU, which can be explained by the obscured symptoms of MH in patients undergoing CPB and the concomitant hypothermia. There is also a 50% probability of the recurrence of MH symptoms after arrival at the ICU, which can be an additional reason for the late manifestation of MH. 

In a systematic review of MH symptoms during CPB, performed by Metterlein et al.,^5^ 14 MH cases were reported from whom, only 5 cases were documented by the caffeine halothane contracture test. We found 11 cases of MH from the review by Metterlein et al. in the cardiac surgery literature: 4 of these cases had a positive contracture test.^11^^-^^20^ We defined 2 important findings in these 11 cases:

- There were delayed diagnoses of MH in the ICU in 6 patients.

- Three of the 4 documented cases with the caffeine-halothane contracture test had late manifestations.

 In the Kriklin/Barratt-Boyes Cardiac surgery textbook, a severe hyperthermia episode during the first 6 to 36 hours after cardiac surgery with an unknown etiology has been described and is assigned to hypermetabolic status. This status has been discussed as a result of CPB, and hypothermia has been recommended as its treatment.^25^ Hypermetabolic state could be a manifestation of MH in which symptoms are masked with hypothermia during CPB and due to delayed diagnosis, it does not respond to treatment with dantrolene.

Apart from the delayed onset of symptoms, which makes the diagnosis of MH difficult, most of the MH symptoms can be mistaken for the complications of cardiac surgery. For example, hypotension and renal failure in MH could be mistaken for the manifestation of cardiogenic shock. Hypoxemia could be mistaken for pulmonary edema as a result of heart failure or pulmonary complications following CPB. Lactic acidosis could be a manifestation of cardiogenic shock as well as MH. Sinus tachycardia and arrhythmia are common in both postoperative phase and MH. Fever could be a manifestation of MH, systemic inflammatory response syndrome during CPB, sepsis, and blood transfusion reactions. Muscular spasms as a sign of MH could be mistaken for convulsion and stroke following cardiac surgery. Urine discoloration secondary to rhabdomyolysis in MH could be mistaken for hemoglobinuria due to hemolysis after CPB. It is rational to consider MH as a differential diagnosis in the following conditions after cardiac surgery:

1- Inability to wean the patient from CPB without a definite cause^14^

2- Oliguria or anuria with concurrent myoglobinuria

3- Unexplained lactic acidosis

4- Unexplained hypotension and sinus tachycardia after cardiac surgery in spite of optimal ventricular contraction and warm extremities, with temporary improvement with fluid and inotrope administration

 Marked increase in CPK as a characteristic sign of MH can be mistaken for perioperative myocardial infarction or related to cardiotomy during cardiac surgery. In 1 of our 2 patients, CPK and CPK-MB were measured and were significantly high without a known reason.

Sedation with benzodiazepines can obscure muscular spasm. In our first case, who did not receive benzodiazepine, muscular spasm continued up to 1 week after surgery. Our second case, however, who received benzodiazepine, did not show any muscular spasm. 

Another limitation in the diagnosis of MH in cardiac surgery is the limitation of capnography, which cannot be used during CBP except for a short period of time at the beginning and at the end of the surgery. 

High PCO_2_, as a diagnostic criterion in MH, is corrected by increasing gas flow by the perfusionist during CPB. In our cases, an increase in PCO_2_ during the postoperative course was not detected in the ICU due to the spontaneous hyperventilation of the extubated patients as well as an increase in the respiratory rate during the mechanical ventilation of the intubated patients.

The CBP setting in cardiac surgery makes an early diagnosis and timely treatment more difficult than in other settings. Classic MH symptoms are obscured and can be misinterpreted as cardiogenic shock, transfusion reactions, and sepsis. Limitations in capnography and paraclinical tests in the diagnosis of MH in cardiac surgery contribute to delayed or even misdiagnosed cases, which should be considered in both of our cases.

Our study has some limitations. The first limitation is the low number of studied cases, which is in part due to the low incidence of MH. As a second limitation, definite diagnosis with the caffeine halothane contracture test was not performed because of its unavailability. Another limitation, which was because of the retrospective nature of the study, was that the diagnosis of MH was not made and dantrolene was not administered so the reversal sign of MH with dantrolene was not demonstrated.

Considering the mentioned limitations, the 2 represented cases did not have a definitive diagnosis of MH; however, alongside other differential diagnoses such as sepsis, transfusion reaction, heart failure, and neuroleptic malignant syndrome, the diagnosis of MH should be considered a highly probable differential diagnosis.

Our aim in this article is to emphasize that in critically ill patients during the early postoperative period after cardiac surgery, MH is a strong differential diagnosis. Missing this treatable syndrome as a possible diagnosis will decrease the chance of patients’ survival.

## Conclusion

The classic symptoms of MH could be obscured and be misinterpreted in patients with cardiac surgery, so it is necessary to be cautious about the application of anesthetics that trigger the onset of MH. In the case of a doubtful diagnosis of MH in patients undergoing cardiac surgery, early treatment with dantrolene should begin. The ICU medical team including the cardiac surgeon, cardiologist, and anesthetist should be aware of the side effects of the trigger substances and their delayed manifestations. Performing capnography measurements routinely in the ICU could be a helpful diagnostic tool for the early detection and treatment of cases with suspicious symptoms of MH in the ICU.

## Notes:

***This paper should be cited as: ***Neshati M, Azadeh M, Neshati P, Burnett T, Saenz R, Karbasi B, Shahmohammadi G, Nourizadeh E, Rostamzadeh M. Malignant Hyperthermia: Report of Two Cases with a Neglected Complication in Cardiac Surgery. J Teh Univ Heart Ctr 2017;12(4):175-183.
